# Student experiences and entrepreneurship education in a specialist creative arts HEI: a longitudinal approach

**DOI:** 10.1007/s41959-022-00085-9

**Published:** 2022-12-09

**Authors:** Nicola Pennill, Keith Phillips, Michelle Phillips

**Affiliations:** Royal Northern College of Music, Manchester, UK

**Keywords:** Entrepreneurship education, Enterprise, Creative arts, Competencies, Career development, Inspiration, Values

## Abstract

A longitudinal study of music students in a specialist higher education institution explored career intentions of students preparing for a career in the music industry. Stage of study had an impact on career goals, with students likely to gravitate away from aspirations for a performance career, towards a portfolio, freelance career. Alumni data supported this, with 80.3% of graduates from the institution currently engaged in a portfolio career. Reasons for changing intentions included gaining awareness of alternatives, personal insights, and negative prospects of the sector. Role models and other sources of inspiration were also key. Early students, especially those holding offers and yet to start, were particularly susceptible to external inspiration, such as advice or experiencing an exciting performance. Alumni were inspired by continuing to learn new skills, and networking with peers. Students were asked to self-rate themselves against generic industry competencies, and were most confident in setting goals and working as a team, and least confident in applying specific business skills such as managing finances. Findings are discussed in relation to the role that entrepreneurship education can play in supporting the needs of students in specialist performing arts, underpinning the need for early intervention and support as students perspectives emerge and are re-evaluated during their studies.

## Introduction

Creative arts graduates face increasing challenges in establishing viable livelihoods, and this is likely to continue to be a difficult industry to enter in the wake of the COVID-19 pandemic and its impact on the arts industry. In this article, we explore some of the themes and findings from a study conducted in a specialist creative arts higher education institution (HEI). In pursuing this research, we consider the forms which entrepreneurship education takes in this setting, and the experiences of students engaging with it and seeking to find and prepare for their place in their chosen field of work.

For the purposes of our study, we adopted the following definition of entrepreneurship, which is widely used in the literature;

“Entrepreneurship is when you act upon opportunities and ideas and transform them into value for others. The value that is created can be financial, cultural, or social” (FFE-YE, 2012, p. 11).

This definition aligns with Scott (2012), who posits that artistic entrepreneurship consists of identifying opportunities that allow for artistic and vocational development, in order to create an innovative product or to generate value. It also builds on the definition proposed by Alvarez and Barney (2007) which suggests that entrepreneurship translates into actions aimed at the creation/discovery of entrepreneurial opportunities. Both assert a connection between artistic action and translation as part of the realisation of value in the world.

Also of relevance here is consideration of ‘enterprise’ which is also subject to multiple definitions. The Quality Assurance Agency for Higher Education (QAA, 2018) further defines enterprise as:

‘The generation and application of ideas, which are set within practical situations during a project or undertaking. This is a generic concept that can be applied across all areas of education and professional life. It combines creativity, originality, initiative, idea generation, design thinking, adaptability and reflexivity with problem identification, problem solving, innovation, expression, communication and practical action’. (p. 7)

In the creative sector, various forms of freelance enterprise are extremely common. For musicians, it is quite usual to combine multiple strands of paid activity, which may incorporate teaching and performing, either with or without some form of employment. Such career choices for musicians are not without their pressures and ambiguities (Cottrell, 2002; Lundin, 2022), and arts graduates generally face the prospect of entering a workplace where co-production, venture creation and portfolio careers are the norm (Bennett & Bridgstock, 2015; Bridgstock, 2013; Damásio & Bicacro, 2017; de Reizabal & Gomez, 2020).

Despite this being a well-established pattern of career development in the creative industries, engagement of students in entrepreneurship education is widely recognised as a challenge. In itself, lack of engagement has been shown to be a result of a perception of students that entrepreneurship education is not relevant for students of creative arts subjects (Clews & Clews, 2011), which in turn may be a reflection of a lack of understanding or awareness of how it links to existing skills. There can also be a resistance from arts academics and students due to a mismatch between their perceptions of the ‘entrepreneurial mindset’ as business-oriented, and the pursuit of art for its own sake (Bridgstock, 2013; Jen, 2019). Perceived access may also be a barrier for some students, as entrepreneurship education may not be seen as inclusive to all (Carter et al., 2015). There can also be a tension between an idealised Bohemian artistic identity, and a professional identity as a self-employed freelancer (Eikhof & Haunschild, 2006; Schediwy et al., 2018a, 2018b). However, when entrepreneurship education is positioned as a way of increasing agency, engagement with entrepreneurship can be seen as a route to empowerment (Bacigalupo et al., 2016). Moreover, such positioning allows for a recognition that these skills are not only required by arts graduates in order to establish portfolio careers (Bartleet et al., 2019; Latukefu & Ginsborg, 2019; López-Íñiguez & Bennett, 2020), but also that they are intrinsic to artistic behaviour itself.

Whilst there are many excellent examples of good practice in entrepreneurship education in the arts, issues with provision in higher education remain. This is in the context of wider change within higher education (HE) in the arts, which has been considerable over the last few decades, driven by a recognition for the need to equip students for the increasingly fluid and uncertain situation they will face after graduating (Brook & Fostaty Young, 2019; Lingo & Tepper, 2013; López-Íñiguez & Bennett, 2020; Patrick et al., 2008; Smith, Bell, Bennett, & McAlpine, 2018). This change is driven by governments and creative industry stakeholders keen to enhance the employability of graduates, and by academics and students who increasingly recognise the importance of transferable skills. There has been a degree of reform in HE, with a greater emphasis on experiential learning methods to promote generic competencies such as problem solving and creativity (Bennett et al., 2017; Brown, 2016; Dhawad, 2015; Harrison & Grant, 2016; Jackson, 2015; Lucas & Tan, 2007; Nielsen et al., 2018; Römgens, Scoupe, & Beausaert, 2019; Zelenko & Bridgstock, 2014). However, gaps still remain in the offer that degree programmes (in both their in- and extra-curricular content) make to creative arts students, in terms of how well they prepare them for their future careers.

Schediwy et al., (2018a, 2018b) follow Bridgstock (2013) and suggest three areas for arts entrepreneurship education. The first, closest to the traditional business school model, entails teaching students how to start up a business: ‘new venture creation’. The second promotes qualities such as scanning the environment for opportunities and being proactive and risk-taking: ‘being enterprising’. The third, ‘employability and career self-management’, involves providing students with opportunities to engage with the artistic labour market, consider its challenges and develop the means to manage a fulfilling career. The QAA (Penaluna & Rae, 2018) advocates the need for degree programmes to include ‘learning about’ (theory), ‘learning for’ (preparatory activities), and ‘learning through’ (in action), which ‘is primarily a reflective process, where a student engages in entrepreneurial activities and maps their own learning and (supported) progression’ (Penaluna & Rae, 2018, p. 14). Building on this, recommendations for HEIs designing entrepreneurship programmes in music have been put forward by de Reizabal and Benito Gomez (2020):

- Ensure clear communication [to students] of all opportunities and how they could support professional development (not just what is happening but why it could be useful)
- Recognise students' 'creative core' and help them recognise their identity, motivations and potential early on
- Help students recognise their value and the many forms it takes, including financial
- Provide opportunities for connecting with people–through working with peers and team working, networking, and meeting inspiring role models

They further recommend care with terminology, use of mentorship and co-creation, and a mix of fun activities and events such as personal development ones (e.g. envisioning future headlines), and idea development (e.g. concept creation, problem solving).

This is closely aligned to the recommendations of Soutaris et al. (2007), who propose that ‘good practice’ programmes offer activities grouped under four components:

- A ‘taught’ component, with one or more modules;
- A ‘business-planning’ component, which can include business plan competitions and advice on developing a specific business idea;
- An ‘interaction with practice’ component, which can include talks from practitioners and networking events; ​
- A ‘university support’ component, which can include market-research resources, space for meetings, a pool of technology with commercial potential and even seed funding to student-teams.

Emerging professional identities are a key part of the learning process. There is an underlying tension between the Bohemian artistic and professional freelancer identities (Gotsi et al., 2010; Schediwy et al., 2018a). Bridgstock (2013) draws on this concept of identity-adaptability to develop the notion of value-congruence. This means that artists can follow their creative motivations whilst considering how to add social, cultural or economic value by sharing what they do with wider audiences in ways which do not conflict with their artistic ideals. In forming their professional identities, therefore, arts students are often required to reconcile a number of contradictory factors. Arts graduates entering the workplace also exhibit motivations that are demonstrably different to other sectors; Bloom (2020) reports that arts graduates are more likely to choose to work in their sector, and prepared to earn less than non-creative graduates. These differences may be attributed in part to a high degree of motivation of graduates driven by the need for value-congruence (Bridgstock, 2013).

In motivating action in working towards life or career goals, inspiration is a key driver, as in turn it impacts attitudes and intentions. As a tool for education, inspiration can be a powerful lever, if designed purposefully to foster an entrepreneurial mindset (Soutaris et al., 2007). The early years of higher education are particularly key in this regard, and can shape future intentions (Nabi et al., 2018). The development of mindset and intentions embraces much more than questions of finding employment and making money, but also personal and professional identity, purpose, motivation and agency; awareness of societal changes; artist agency and impact; ideation, finding new opportunities, adapting; and being able to collaborate (Gaunt, 2018).

Specific skills and competencies are also key when designing and evaluating programmes of education. The QAA, 2018 guidance draws a distinction between enterprise and entrepreneurship, in which ‘enterprise’ comprises generic skills and attributes (see definition, above) and ‘entrepreneurship’ relates to the application of these enterprise attributes and skills in behaviours that create value (cultural, social, economic), and is not limited to new venture creation. The UK Professional Standards Framework (Advance, 2019) is based on the QAA guidance, but sharpens this distinction by limiting the scope of entrepreneurship to new venture creation. The question of which competencies constitute ‘core’ entrepreneurial competencies has been the subject of much rigorous research and exploration. Most notably, the EntreComp framework (Bacigalupo et al., 2017) seeks to place these skills within a comprehensive context relevant to a wide range of settings and has become the de facto source for more specialist iterations. Based on this and further work within higher education, the QAA Framework was developed in 2018 to support learners and educators in delivering consistent outcomes among students. In music higher education, a pan-European project of music conservatoire partners aimed to promote entrepreneurship as a component of higher music education (HME) programmes. This project (RENEW: Reflective Entrepreneurship Music Education Worldclass) identified and categorised ‘hard’ and soft’ skills as they apply to the musician entrepreneur (de Reizabal & Gomez, 2020). A further principle at play here is the extent to which music students engage with the concept of entrepreneurship, as discussed above, and how this relates to their confidence in their capabilities as it relates to their own goals. In her work with music students, Jen (2018) found this to be a critical element for engagement with entrepreneurship training.

In order to support students in their learning, a bespoke approach is required, to adapt competencies and delivery to be relevant and focussed for the music profession. Laying out the findings of the RENEW project, Gaunt (2018) made this point strongly when reflecting on the requirements for music higher education:

‘...Simply importing generic entrepreneurship modules, as developed for example in business schools, is unlikely to be successful’ (p. 1).

Recommendations for effective entrepreneurship education for musicians include the importance of identity and mindset development (de Reizabal & Gomez, 2020), the use of role models, mentoring, creating 'visions of possible selves', not over-emphasising business skills, confidence building, and addressing fear of failure (Jen, 2019).

### Gaps and approach

Given the increasing challenges faced by the cultural sector, and in order to better understand some of these barriers, the underlying goals of this research were to explore how students can be supported to develop the skills and knowledge required for a career in the creative industry by understanding career intentions, sources of inspiration and students’ level of confidence at different stages of study. This article outlines some of the barriers and initiatives identified within specialist higher education, and proposes ways to foster greater access to entrepreneurship skills and different forms of creativity in students. Worldwide, more than 10 million jobs were lost in 2020 due to the pandemic (Sherwood, 2022). In 2019, the DCMS estimated that the creative industries contributed £115.9 billion to the UK; 5.9% of the UK economy. Between Q4 2019 and Q2 2021, the sector’s output declined by 37%. Nurturing the creative professionals of the future is vital to recovery, and to ensure a thriving and diverse society. Arts graduates are often highly committed to work in their chosen field of practice (Bloom, 2020), but need support and training to establish sustainable careers and to bring their creative visions to life. In seeking to address this, a two-year study (the StART Entrepreneurship Project) funded by the Office for Students and Research England between three arts higher education institutions (HEIs) explored the issues, barriers and needs of students as they transition into the world of work.

The Royal Northern College of Music (RNCM, project lead), the University of the Arts London (UAL), and the Royal Central School of Speech and Drama (RCSSD) engaged with current, past and future students to understand their needs and to design teaching interventions and resources. In this study, we report on findings from the RNCM in which an in-depth longitudinal case study explored the following themes:

- Changes in career aspirations which happen at different stages during students’ courses of study, and what might impact those changes.
- Self-assessment of core entrepreneurial competencies, particularly focussing on transferable skills which cross creative domains.

We were also keen to make use of an opportunity to adopt a longitudinal approach to track student journeys; as highlighted by Nabi et al. (2017) in their review of the impact of entrepreneurship education, much (68%) of the existing research makes use of cross-sectional survey methodology. The authors also highlighted the dearth of empirical data on how intention translates into action, the importance of transition-based indicators, and how these are impacted by entrepreneurship education. Whilst this study does not track students over an extended period, the two-year timeframe allowed for investigation of several key transitions.

The following research questions were investigated:

1. How do career intentions, confidence in entrepreneurship competencies and sources of inspiration develop and / or vary over time?
2. How do students describe the reasons for changing career intentions during their undergraduate studies?

Interventions were designed which built on existing provision and incorporated good practice drawn from the literature and wide consultation with industry and educators. The RNCM degree programme consists of three parts in each of the four years of study: (1) Academic studies (music theory, history, and specialist options such as music psychology), (2) Professional skills training (termed ‘Artist Development’), and (3) Principal Study (vocal, instrument or composition specialism). All three parts are weighted almost equally, and Principal Study being responsible for slightly more credits than the other two areas. Students acquire 120 credits per year in each of the four years of study. The parts of the undergraduate degree programme and annual schedule at the RNCM which were developed as part of the StART programme and hence relevant for the current study were:

- Year 1–Artist Development 1 module (20 credits), redesigned for 2022 as part of the BMus degree programme revalidation
- Year 2–Artist Development 2 module (20 credits), redesigned for 2022 as part of the BMus degree programme revalidation
- Year 3–Professional placement module (2021 and 2022, 20 credits), redesigned to include and maintain an in-house element during and following the COVID-19 pandemic
- Year 4–Extra-curricular one-to-one career interviews between students and tutors (2021 and 2022)

All RNCM students were also eligible to opt into specialist entrepreneurship coaching (‘StART Conversations’) to develop their ideas, and to take forward ideas to the RNCM Creative Innovators Award for entrepreneurship (an annual extra-curricular award with a prize fund of up to £2,000 per student plus industry mentoring). However, it should be noted that data collected for the current study with RNCM students includes their reflections and responses in the context of their entire degree experience, and not just the activities considered to represent entrepreneurship education.

## Materials and methods

Data were collected over an eighteen-month period. The research was designed to sample students who were at five different stages of their degree programmes (offer holders, Y1, Y3, Y4, post-graduation), and from two consecutive year group cohorts (2020–21 and 2021–22). Music conservatoire degrees in the UK are usually three years (rather than three, which is the norm for university-based degree programmes). Data were not collected from year 2 students as the project focussed on changes in career intentions from the beginning of a student’s degree programme journey (year 1, Framework for Higher Education Qualifications of UK Degree-Awarding Bodies (FHEQ) Level 4) and later stages (years 3 and 4, which both form Level 6 study, which is the level at which credits contribute to the degree classification). Student surveys were distributed at regular intervals during the academic year to explore themes relating to the core research aims. Specifically, the surveys focussed on career aspirations, how these changed over time, and what support students felt that they needed as they moved through their course of study. Based on feedback from year 4 students, music therapy was added as a career option after the first round of survey data was collected. Outlines of surveys for offer holders, enrolled students and alumni are included in the Appendix.

### Data sources, instrument, and measures

Primary data were used for this study. Likert scale and open text responses were used to elicit self-reported data on career intentions, sources of inspiration, and confidence in core entrepreneurship competences. Basic demographic data were also collected, which included gender and study specialism (e.g. composition, voice, instrument, popular music).

The questionnaire was piloted with a cross-section of students before being finalised. Prior to data collection, ethical approval was obtained from the RNCM Research Ethics Committee, and individual consent was sought from all respondents. The questionnaire took 15–20 min to complete. Each item was measured on a seven-point Likert scale ranging from ‘Strongly Disagree’ to ‘Strongly Agree’ with scores from 1 to 7 correspondingly. The questionnaire was designed to elicit information on the three overall constructs, namely career intentions (13 items), sources of inspiration (10 items), and confidence in competencies relating to skills in entrepreneurship (13 items). Questions were drawn from the literature and validated for relevance and meaning through piloting with staff and students.

### Data analysis

Frequencies and percentages were used to describe the distributions across the study population. Quantitative analysis used Mann–Whitney U tests for between-group comparisons and Wilcoxon signed ranked tests with Bonferroni corrections for within-group comparisons. Qualitative data were analysed using thematic analysis. Responses to the three open text questions from the survey were analysed, from students currently studying on degree programmes (years 1, 3 and 4). Similar data were collected from offer holders and alumni in order to provide points of comparison prior and post participation in the degree programme.

The open text questions posed were:

1. What advice would you give other students in relation to achieving future career goals?
2. What advice or support do you need now?
3. What are the reasons for changing your ideas about your future career since starting at RNCM?

### Research design, population, and sampling procedure

The research adopted a mixed methods approach, based on a sequential explanatory research design to examine the research problem at a number of points over time (Creswell and Creswell, 2017). Mixed quantitative and qualitative data were collected using self-report surveys of students, according to the schedule outlined in Table 1. The questions were designed following themes from the literature, and refined based on feedback from staff and students during a pilot phase.

**Table 1:**
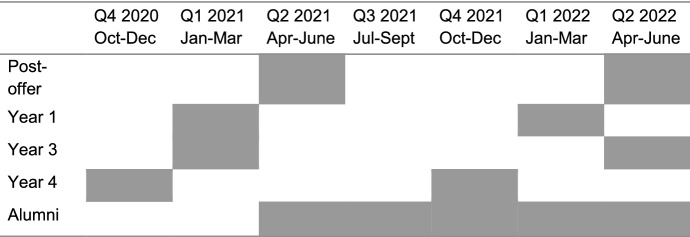
Timeline of data collection for current students in year groups 1, 3, and 4, post-offer, and alumni

The approach aimed to capture the extent to which events, people and experiences have influenced current ideas of future work (‘sources of inspiration’), the degree of confidence in applying core competences, and how career intentions have changed from the start of students’ study to their current stage. Sources of inspiration were adapted from Nabi et al. (2018). For career intentions, students were asked to recall their aspirations on joining the degree programme, and to report their current intentions. (This information was collected alongside current career intentions based on students’ *recollection* of career intentions on entry to a degree programme, rather than data captured at the time when they actually began their studies).

A list of competences was drawn from the QAA framework (Penaluna & Rae, 2018), and students were asked to self-assess their current level of confidence in applying each. Students were also asked to reflect on particularly influential or memorable events or people that had helped form their intentions to date.

Eligible participants comprised those students enrolled on or holding a current offer for the BMus programme at the Royal Northern College of Music, as well as graduates (alumni) of the college, from any previous year of study. Questions were adapted to ensure they were appropriate and meaningful for each year group. The surveys were issued to the target groups of participants who then had 2–6 weeks to submit responses, except for the alumni survey which remained open from March 2021–June 2022. A total of 401 responses were received, and 395 responses validated. Of these, 217 (54.9%) were female, and 165 (41.8%) male. A further 9 (2.3%) preferred not to say (PNTS), and 4 (1%) classified themselves as non-binary. The responses represented a relatively well-balanced cross-section of specialist disciplines (composition, voice, instrumental study or popular music) (see Table 2), although when considered across cohorts, the majority of respondents (69.4%) were from the schools of wind, brass and percussion, vocal studies and strings. (Table 3).

**Table 2:** Number of respondents by year group, indicating distribution of gender (female, male, prefer not to say, other)

	OH21	OH22	Alumni	Y1 2021	Y1 2022	Y3 2021	Y3 2022	Y4 2020	Y42021	Total	%
Female	13	35	46	19	5	28	18	37	16	217	54.9
Male	18	38	22	24	6	17	8	23	9	165	41.8
PNTS	1	2	3	1	0	1	0	0	1	9	2.3
Other	0	1	1	0	0	1	0	0	1	4	1.0
Total	32	76	72	44	11	47	26	60	27	395	100.0

**Table 3:** Number of respondents by year group, indicating distribution of school of study (specialist discipline)

		OH21	OH22	Alumni	Y1 2021	Y1 2022	Y3 2021		Y3 2022		Y4 2020		Y42021		Total		%
Strings		7	17	10	6	2	9		9		13		8		81		20.5
Wind, brass, percussion		10	13	36	4	3	9		9		16		11		111		28.1
Voice and opera		6	12	4	4	2	26		7		16		5		82		20.8
Keyboards		4	13	12	1	2	1		0		7		2		42		10.6
Composition		5	6	7	1	2	0		1		5		0		27		6.8
Popular music		0	15	2	28	0	2		0		3		0		50		12.7
Other		0	0	1	0	0	0		0		0		1		2		0.5
Total		32	76	72	44	11	47		26		60		27		395		395

## Results

Our research questions were as follows:

- How do career intentions, confidence in entrepreneurship competencies and sources of inspiration develop and / or vary over time?
- How do students describe the reasons for changing career intentions during their undergraduate studies?

Results are therefore presented and discussed in two parts:

1) Development of career intentions, including sources of inspiration and reflections on experiences, and 2) Confidence in generic competencies.

### Changing career intentions

**Fig. 1:**
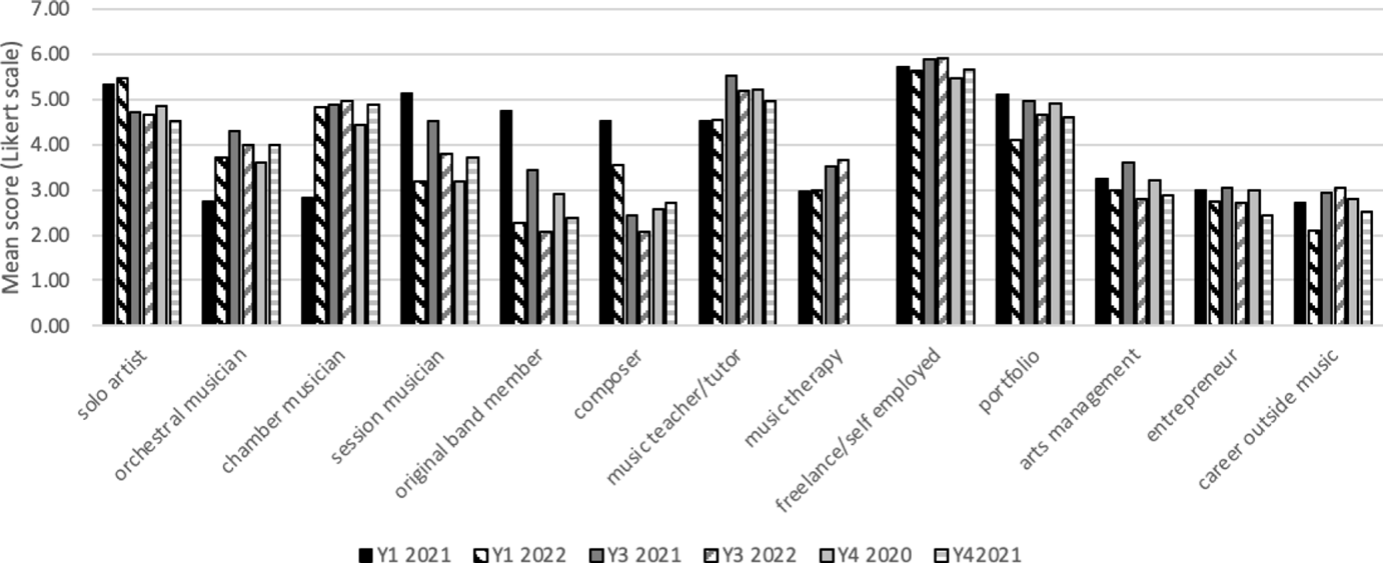
Recall of career intentions at start of studies for students currently studying on degree programmes (Years 1, 3, and 4). Year groups are shown as the year in which students completed the surveys, i.e. ‘Y1 2021’ represents students in year 1 of their studies in the academic year 2020–21

**Fig. 2:**
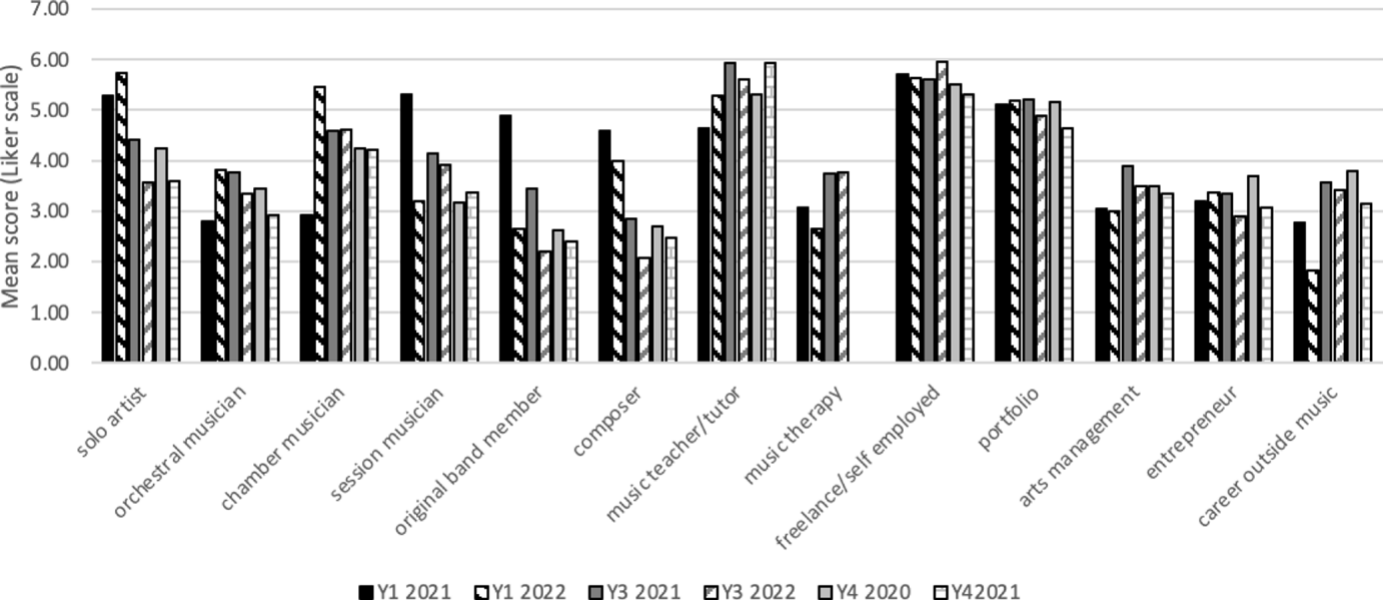
Career intentions for current students (Years 1, 3, and 4), at time of data collection

When comparing career intentions at the time of sampling with recall of intentions at the start of a student’s programme of study, career intentions had changed over this period. Specifically, intentions tended to gravitate away from ‘independent performer’ (which included the categories ‘solo artist’ and ‘band member’) and towards more portfolio careers in which performance was a smaller part, and other sources of income were also part of the career. This effect was more pronounced for students in later year groups—students in both years 3 and 4 showed significant differences between recall of aspirations on joining their degree programme and at time of survey (i.e. 2.5–3.5 years later). Wilcoxon signed-rank tests showed that for year 4 students there were significant decreases in intention to pursue careers as a soloist (*T* = 91.0, *p* = 0.003, *r* = 0.27) and original artist (*T* = 50.5, *p* = 0.021, *r* = 0.21) and increases in intention to become an entrepreneur (*T* = 453.0, *p* = 0.007, *r* = 0.24) and to have a career outside music (*T* = 529.4, *p* < 0.001, *r* = 0.37). In year 3, there was also a significant shift away from an intention to pursue a solo career (*T* = 48.0, *p* = 0.045, *r* = 0.21) and towards a career outside music (*T* = 34.5, *p* = 0.012, *r* = 0.26). In contrast to the change in intentions towards a career related to entrepreneurship in year 4, year 3 students showed a significant shift towards a teaching career (*T* = 165.5, *p* = 0.003, *r* = 0.31). For year 1, there were no significant differences, i.e. students career intentions were not found to have changed significantly between beginning their studies, and whilst part way through their first year of study. (Figs. 1 and 2).

**Fig. 3:**
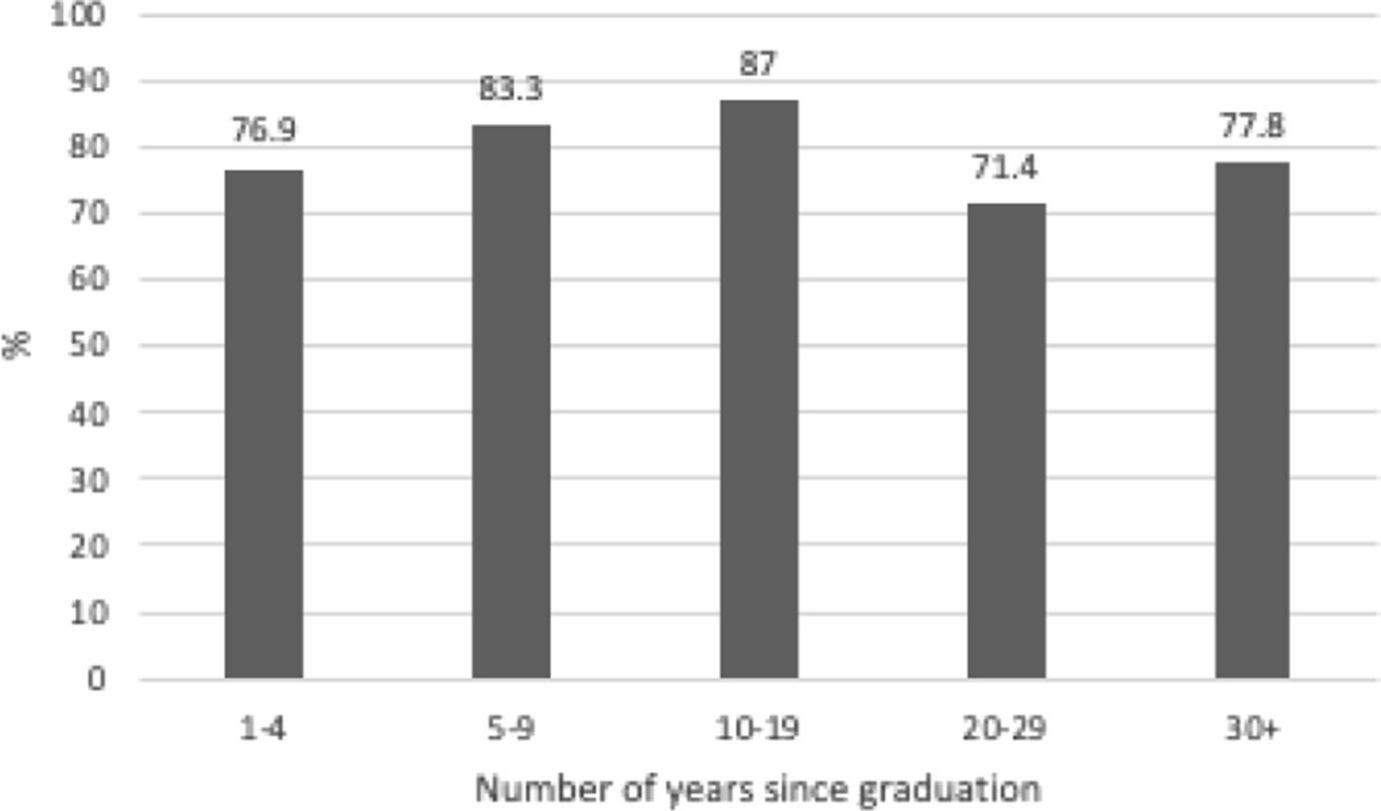
Percentage of graduates pursuing a portfolio career with multiple roles, by years since graduation

A survey of alumni captured data from graduates spanning the years 1975–2022. Alumni were asked whether they currently have a portfolio career with multiple roles (e.g. some performing, some teaching, for example). There were 71 valid responses, of which 80.3% answered ‘yes’ to having a portfolio career. The distribution of respondents according to years since graduation was as follows; 1–4 years (*n* = 13), 5–9 years (*n* = 12), 1019 years (*n* = 23), 20–29 years (*n* = 14), and 30 + years (*n* = 9). The percentage of those pursuing a portfolio career at the time of data collection is summarised in Fig. 3. This data indicates that, from the very beginning of their careers, a significant proportion of graduates transition into a portfolio career. Those graduates with more established careers (5–19 years post-graduation) most frequently reported that they fulfil multiple roles, when compared with early (1–4 years post-graduation) or later (20 + years).

Reasons for changing career aspirations over the period of degree programme studies were analysed thematically. The main reasons given were that the change happened because of:

- Graduates acquiring new information or knowledge
  - Being shown new paths and career directions
  - Recognising the need for more training or support
  - Recognising what is required to achieve success and adjusting expectations
- Graduates gaining new personal insights
  - Clarifying own interests and strengths and bringing new areas into focus
  - Changing personal values or circumstances
- Changing career or market prospects
  - Graduates may be less confident in music as a career because of uncertainty or lack of suitability
  - Graduates may be looking at other careers

The data collection period coincided with the COVID-19 pandemic, in which the cultural industries were significantly impacted in the UK and beyond (Comunian, 2020). This additional uncertainty in the industry may have impacted the overall confidence of students in the future of music as a profession. The importance of career-focussed interventions (which might be offered as in- or extra-curricular within a degree programme of study) was underlined here, as students adjusted their plans based on what they learned during their studies. This applied across all year groups (1, 3 and 4), for example;

“As I have learnt more about the industry as a whole, wider career opportunities have presented themselves to me.” Year 1 student, 2020-2021 cohort

### Sources of inspiration at different stages of study

**Fig. 4:**
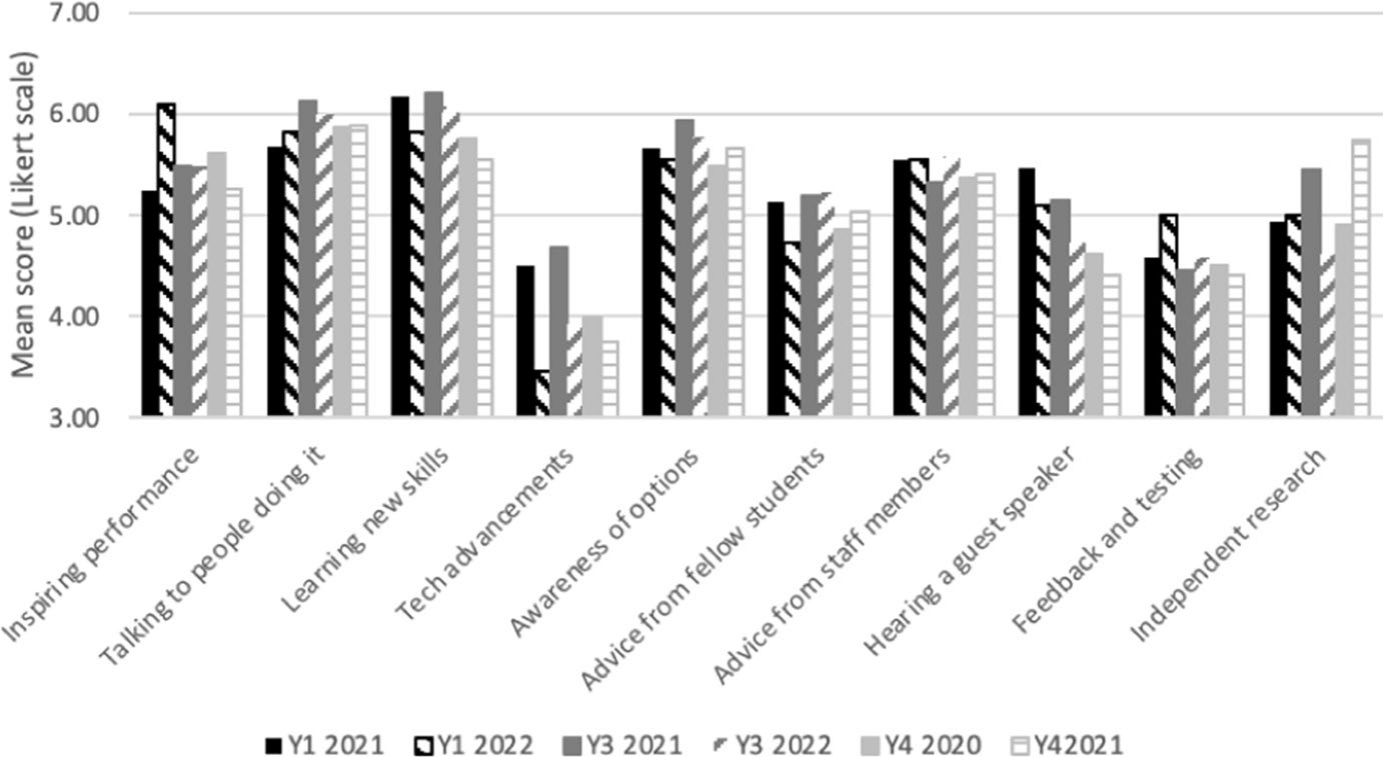
Sources of inspiration by year group for students currently studying on degree programmes

Students studying for their degree programme (i.e. not including offer holders or alumni) cited sources of inspiration as mostly experiential–performances seen, the learning of new skills, and talking to people working in the music industry. Less inspiring were technological advancements and getting feedback following testing out ideas. Guest speakers were most inspirational for students in the earlier stage of study (year 1) (see Fig. 4). As with the findings of Nabi et al. (2018), who also explored the impact of interventions with year 1 students in higher education, and found that a combination of theoretical learning, practical, hands-on experiences, and strong inspiration from peers and role models were especially impactful. These are all elements which can be readily facilitated through entrepreneurship education.

Comparing mean scores of most common sources of inspiration, between current students, alumni and offer holders (see Table 1), it was notable that offer holders were generally more susceptible to external sources of inspiration. In particular, offer holders were most inspired by advice from others, for example staff and fellow students, or by experiencing an inspiring performance. Alumni continued to be inspired by learning new skills and talking to others in similar fields. (Table 4).

**Table 4:** Sources of inspiration for offer holders, current students and alumni

Sources of inspiration	Mean score from Likert scale (1–7)		
	Offer holders	Current students	Alumni
Experience of a performance	6.28	5.25	5.83
Talking to people doing it	6.03	5.68	6.10
Learning new skills	6.00	6.18	6.00
Tech advancements	4.37	4.50	2.74
Awareness of options	5.53	5.66	5.19
Advice from fellow students	5.79	5.14	4.68
Advice from staff members	5.94	5.55	5.51
Hearing a guest speaker	4.40	5.48	4.67
Feedback (on ideas) and testing	4.79	4.59	4.38
Independent research	5.51	4.93	4.50

### Confidence in competencies

Overall, there was a wide variation in confidence in core competencies between students, and between competencies (see Fig. 4). The competencies analysed were:

- Identifying employment or business opportunities
- Making choices about which opportunities to pursue
- Making contact with new people
- Setting own goals
- Using digital, data and media to support my work
- Starting and leading projects
- Managing finances
- Creating a business plan
- Managing risk
- Working in a team

**Fig. 5:**
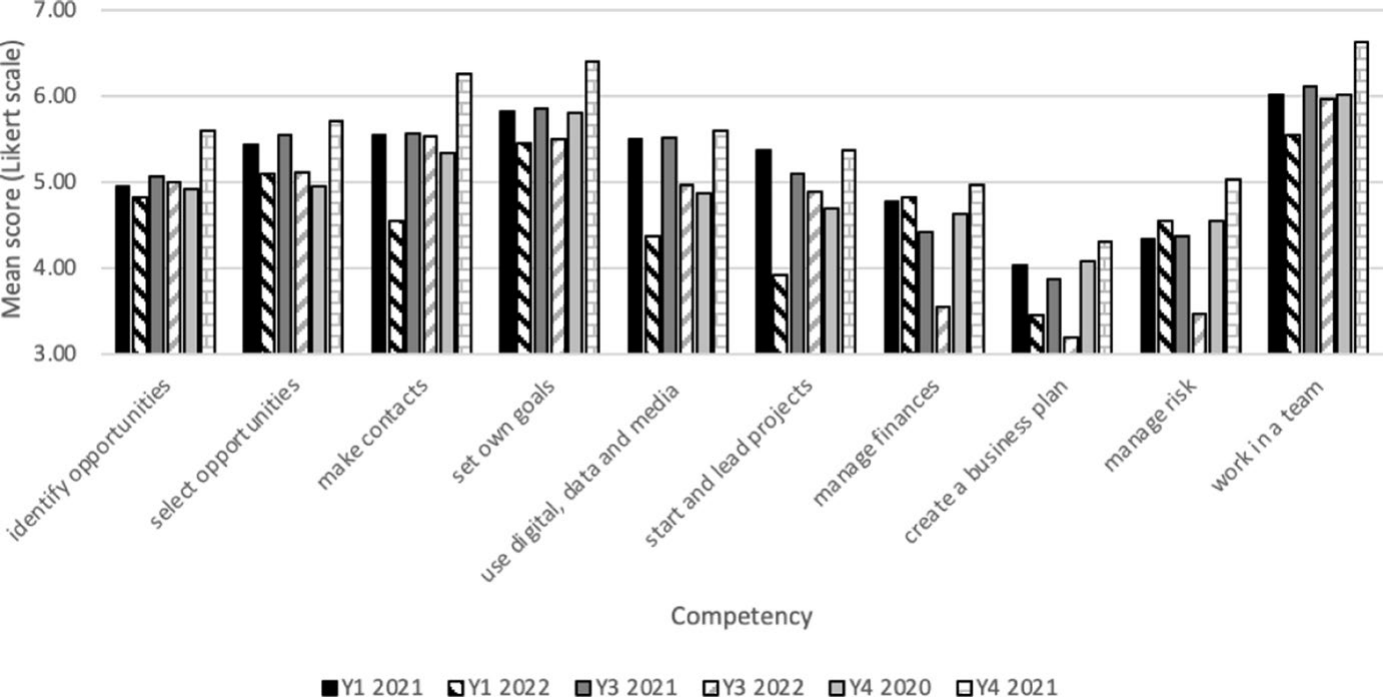
Confidence in core competencies for current students (Years 1, 3 and 4)

Students were most confident in setting goals and working in a team, and least confident in applying specific business skills such as managing finances (see Fig. 5). Comparing consecutive year groups, the year 1 students in 2021–2022 were statistically less confident than the 2020–2021 year 1 cohort in making contact with new people (*p* < 0.05), and using digital technologies (*p* = 0.013). Other competencies showed no difference between year groups or years of study. The newer students (year 1 2021–2022) had experienced their most recent education during COVID-19-pandemic-related remote learning, which may account for being less confident with making new connections, although this does not account for their reporting being less confident with the use of digital technology. Year 3 students in 2021–2022 were statistically significantly less confident in managing finances (*p* = 0.049), and managing risk (*p* = 0.037), than the year 3 cohort 2020–2021. Again, the background of financial uncertainty for the music industry and wider economy due to COVID-19 may account for this. However, year 4 students in 2021–2022 were statistically significantly more confident than the 2020–2021 cohort in making contact with new people (*p* = 0.012), setting their own goals (*p* = 0.044) and working in a team (*p* = 0.036). A key difference between these cohorts was that the 2021–2022 group had experienced focussed training via the redesigned Placement Module within the degree programme, which included a StART Entrepreneurship strand for those that opted in. This finding could therefore reflect the impact of the new in-curricular content established as a direct results of the StART Entrepreneurship Project.

### Reflections from students on formulating career plans

Students were invited to reflect on their reason for changing their career aspirations since joining their degree programmes at the RNCM. There were two main themes emerging from this question; firstly, that their experiences of higher education had changed their perspective on their options, and secondly that they were experiencing a process of re-evaluation driven by personal or external factors. The emergence and importance of personal values as a driver in career choice was further reinforced when students were invited to offer advice to their peers, which focussed strongly on the importance of developing a sense of agency, cultivating a strong work ethic, and building personal networks.

### Emerging perspectives on career aspirations.

An important theme was the emergence or clarification of personal values or attitudes, and development of strengths and interests, as typified by the following comments from final year students:

“Changed my personal values or attitude to my choice of career . Clarified my own interests and strengths to enable me to focus on new areas .”

“I have found that there are certain stresses that come with a job that I don't need or want in my life, other [reasons for changing career direction] are a general lack of compatibility or interest.”

These emerging perspectives both positively and negatively impacted students’ views on their possible futures. For some, it increased their interest and resolve to pursue music, whilst for others it helped to crystallise a realisation that other paths were more appealing. Some students felt they had been shown new paths and career directions, or that their experiences had highlighted areas they had not previously considered.

“Knowing the industry better has made me become more flexible with my future aspirations.” Year 4 student, 2020-2021

“I initially wanted to pursue a career as an original artist but have now decided I wish to be a Music Therapist. My time (at college [RNCM]) allowed me to reflect on my academic intentions and the person I wish to be. I do not think I would cope well with an unstable profession and so decided to pursue something to help others as well as allow me to continue being musical.” Year 4 student, 2020-2021

“The main interests that I have are the same, but I have become more aware of the various opportunities that there are. I’ve had more teaching experience and would love to further explore this avenue whilst pursuing a career in performing. I’m also really interested now in how the general public perceive classical music and I would love to get more involved in community projects as part of my career. This is due to my placement module last year as well as my creative project this year.” Year 4 student, 2021-2022

### Re-evaluation

Closely aligned to these emerging perspectives was a process of re-evaluation. This manifested itself in two main ways. Firstly, a number of students described a loss of confidence in music as a career choice, particularly citing increasing uncertainty in the sector:

“I am now much less drawn to a career in music. This is partly due to lack of confidence …and partly due to my wish to pursue other closely related interests.” Year 4 student, 2020-2021

This lack of confidence was also expressed as the wish to take a break, the need for further reflection, or active pursuit of alternative choices outside music. However, for many, this process of re-evaluation resulted in resolving to pursue their chosen career in music, perhaps by a different path:

“I initially intended to dive into having a professional singing career right after finishing my degree, but I realised that it is more ideal to take a couple of years off to give more time for the voice.” Year 4 students, 2020-2021

Students were also asked what support or advice they needed to achieve their career goals. The main themes which emerged related to the need for signposting and practical career advice; how to get a first job, prepare a CV, get feedback on ideas, find opportunities in the UK and internationally, undertake employment and job-specific training, and find contacts and identify role models and mentors. Also, a number of students wanted support in the form of further training in particular areas (e.g. on managing finances), and/or personal, emotional, or mental health support. Academic support was also requested by some, as they approached important assessments.

### Advice to peers

We also considered the responses of students in relation to advice to their peers. The following themes emerged from thematic analysis of open text responses from students answering the question ‘What advice would you give other students (in relation to career plans)?’. The themes can be grouped into three main categories: self-management, agency and professional skills development.

- Self-management (internal focus):
  - Know your own limits and boundaries
  - Prioritise your own goals and focus on those
  - Accept uncertainty
- Agency (external focus):
  - Be open to opportunities; seize every chance to try something new
  - Be flexible and adapt
  - Ask for help
  - Develop contacts
- Professional skills:
  - Work hard
  - Develop good habits; be prepared, organised and punctual
  - Make use of learning opportunities

Students were very ready to share advice and encouragement with their peers, and also to offer practical suggestions for future degree programme development.

“Discover and see what your fabulous self can do. Being Fabulous can be done in so many different ways...” Year 4 student, 2021-2022

“I believe having a mentorship program would go a long way. I am in need of mentors to whom I can talk to about music. My primary instrument teacher is a wonderful mentor. But his approach is only one. A more ready access to talk to various people and perhaps have a mentor would be quite nice.” Year 1 student, 2021-2022

In summary, our results revealed a complex picture for students, in which they were seeking to advance and hone their professional skills in a highly competitive sector, reconcile their personal identities and goals, and adapt to a rapidly changing workplace which had been severely impacted by the global pandemic. The main themes and findings are discussed primarily in relation to the implications for specialist higher education institutions (such as a music conservatoire) and what educators can do to support future cohorts.

## Discussion

This study collected longitudinal data to examine the topics of career intentions and aspirations of current music students studying on degree programmes, those holding offers to study music in higher education (currently in secondary education, aged 17–18 years), and music graduates (alumni). This study is one of the first to take a longitudinal approach to questions of career intentions, and how these may change over the course of a degree programme of study. Moreover, the current study examined reasons for changes in career intentions, and changes in sources of inspiration for those choosing to pursue a career in the music industry.

The study surveyed offer holders, current students and alumni of classical and popular music degree programmes at the specialist music conservatoire, the Royal Northern College of Music (RNCM). The RNCM has a student body of around 950 undergraduate and postgraduate students, most of whom choose to study music at this specialist institution with the aim of embarking on a career in the music industry. Hence, this environment provides not only an opportunity to study the career aspirations and changes in these for those intending to work in the music industry, but more broadly to examine how students in higher education who enter degree programme study (with one main career intention – to work in the music profession) might change their intentions, and what might impact such changes. The two-year StART Entrepreneurship Project provided an ideal opportunity to ask these questions and survey two cohorts of students (2020–2021 and 2021–2022) in their first year of study, and in their two final years of study (year 3 and 4) as they prepare to enter their chosen careers.

The current study asked the following two research questions:

1. To what extent do career intentions, confidence in entrepreneurship competencies and sources of inspiration develop or vary over time?
2. How do students describe the reasons for changing career intentions during their undergraduate studies?

Results suggested that over time, there was an increase in the number of students who showed an intention to be an entrepreneur or have a portfolio career (undertaking multiple roles in their profession, for example, as a performer and teacher). Year 4 students were significantly more likely to choose a portfolio or entrepreneurial career in this final year, than at the start of their studies. This career intention was also reflected in data from current alumni which showed that, in total, 80.3% were either fully or part time engaged in portfolio careers. Bennett and Bridgstock (2015) reported similar findings in their survey of music and dance students, in which, despite early intentions for a performance career, graduates five years on were mostly engaged in portfolio careers. This discrepancy between expectation and reality remains a core theme for music students and the role of higher education institutions in supporting their needs.

There are a number of wider recommendations that might be made as a result of the findings of this study, which are relevant not only to specialist music study, but to higher education more broadly. Firstly, design of degree programmes needs to account for the way in which student aspirations, intentions, and career plans will likely change during the course of their studies, and should support students in making these decisions, and prepare them for the career that most are likely to embark on, for example if this is a portfolio / freelance career. This notion of preparing students for their careers (for example, the preparation of music students for freelance careers), and supporting them in planning these throughout their course of study, should be foregrounded from the very beginning of their studies, and in- and extra-curricular support should be flexible, where possible, to support changes in such career plans. Students should also be encouraged to develop metacognitive skills, whereby they are taught and empowered to reflect and self-assess skills, and to seek out the information and guidance they need to embark on and shape their career.

Sources of inspiration for current students include seeing performances, learning new skills, and talking to / meeting people in the music industry. Students in their first year of study also found guest speakers to be a source of inspiration. Again, these findings may be useful for the design of degree programmes, both in terms of in-curricular and extra-curricular activities. These findings highlight the importance of key skills training being embedded in a core curriculum, but also the need for institutions to build partnerships with relevant industry contacts who students may work with, perhaps by hearing them as guest speakers, or partners becoming mentors, or offering student placement or shadowing opportunities. Design of key skills learning initiatives may be supported not only by individual subject benchmark statements, but also by the existing QAA (2018) guidance in relation to skills around enterprise and entrepreneurship. Students could also be supported in the learning of key skills by institutions inviting their feedback on the skills which they feel they would like / need to develop. Such skill assessments may be designed with reference to existing frameworks such as EntreComp (Bacigalupo et al., 2017).

Reasons given for changing career intentions include the acquisition of new knowledge or information, new personal insights, and changing markets and prospects (likely linked partly to the COVID-19 pandemic and its aftermath, and this event being at the forefront of students’ mind at the time of data collection). These findings underline the role that the students themselves (and their own personal beliefs, values and artistic identities), the higher education provider (and the knowledge and information contained in a degree programme) and the external industry which students are preparing to enter, play in shaping a students’ career intentions.

**Fig. 6:**
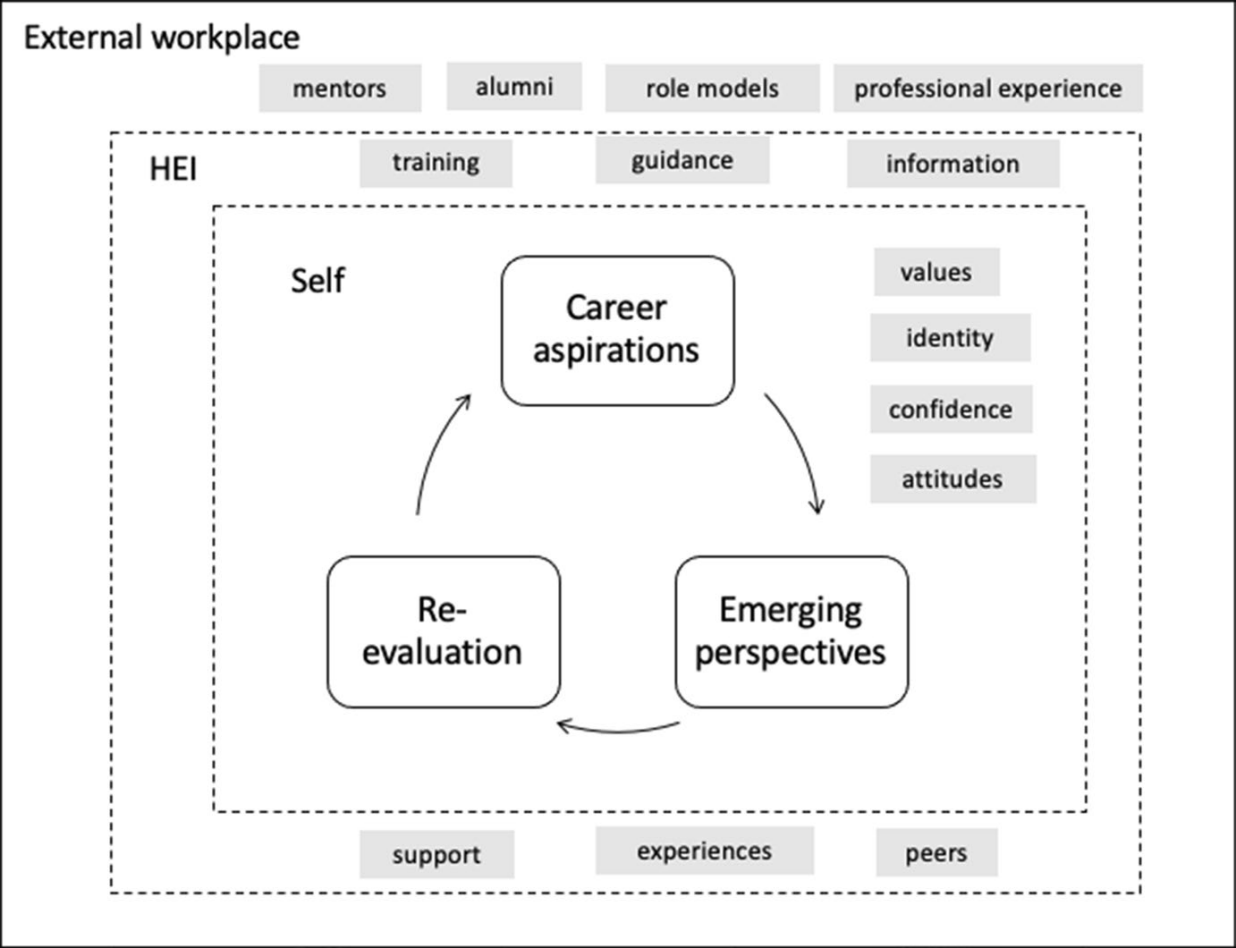
Evolving career aspirations of undergraduate music students

The findings in this study were driven by emerging student perspectives, in which higher education institutions and external potential workplaces (and music industry professionals) have important roles in influencing and inspiring through a framework of support and intervention. The current study demonstrates the impact of this influence reflected in the changing aspirations but also, critically, in the changing values, attitudes and confidence of students as they move through their studies. Figure 6 represents these various levels of influence on a student’s career intentions and aspirations, and emphasises the key parts that the student themselves, the institution, and the external industry play in such decisions and degree programme journeys.

## Conclusions and implications

This study takes a longitudinal approach to the exploration of the career intentions of music students preparing for a career in the music industry, how these change over time (from holding an offer of a place at a music conservatoire for degree programme study, to having graduated), and the sources of inspiration that students find useful in making decisions about their careers over the course of their studies and beyond.

The findings demonstrate that specialist music students’ career plans do change over the course of their studies. Students in the earlier years of study are more likely to plan a solo career as a performer, and students in the later years of study are more likely to intend on a portfolio career which includes multiple roles in the music industry, i.e. working as a freelance musician.

Music students place value on key skills training during their studies, along with opportunities to see performances, and to meet people in the music industry. It is clear that students feel that they do develop certain categories of competencies through their studies. However, with additional support (for example, degree programmes designed with reference to relevant tools and frameworks such as QAA (2018) guidance and EntreComp) and the support to develop metacognitive abilities, students may have even more career options open to them, and agency in their futures.

## Appendix : Outline of survey questions

Thinking back to when you first arrived at RNCM, how clear were your ideas about your future working life? Please indicate how strongly you agree with the following statement, for each of the career choices given. At the start of my studies, I intended to pursue a career as a:

Thinking back to when you first arrived at RNCM, how clear were your ideas about your future working life? Please indicate how strongly you agree with the following statement, for each of the career choices given. At the start of my studies, I intended to pursue a career as a:
